# Supplementary material for: Molecular Genetic Diversity of Local and Exotic Durum Wheat Genotypes and Their Combining Ability for Agronomic Traits under Water Deficit and Well-Watered Conditions
Source: Life (Basel). 2023 Dec 1;13(12):2293. doi: 10.3390/life13122293 (PMC10744700; doi:10.3390/life13122293)
Supplement: Supplementary file 1 [file life-13-02293-s001.zip › life-2695945-supplementary.pdf]

Table S1. Name, pedigree and origin of eight durum wheat genotypes used in the present study.

No	Parent name	Pedigree	Origin
1	W988	ZENIT/5/SORA/2*PLATA-12//RASCON_37/4/ARMENT//SRN_3/NIGRIS_4/3/ CANELO_9.1/6/MINIMUS_4/GRO_2/3/PROZANA /ARLIN//MUSK_6/5/SULA/RBCE_2/3/HUI//CIT71/ CII/4/RYPS27_3/SKARV_3	CIMMYT
2	W994	ARMENT//SRN_3/NIGRIS_4/3/CANELO_9.1/4/STT //ALTAR84/ALD/3/PATKA_7/YAZI_1/5/HUALITA	CIMMYT
3	W996	TRIDENT/3*KUCUK	CIMMYT
4	W1011	CIRNO C 2008/3/KNIPA/TAGUA//PLANETA/TRILE	CIMMYT
5	W1518	COPIO/3/ATTILA*2/PBW65*2//KACHU/4/NELOKI	CIMMYT
6	W1520	KSW/SAUAL//SAUAL/3/TRCH/HUIRIVIS #1/5/UP2338 *2/SHAMA/3/MILAN/KAUZ//CHIL/ CHUM18/4/UP2338*2/SHAMA	CIMMYT
7	Bani-Suef 5	Dipperz/bushen3 CDSS92B128-1M-0Y-0M-0Y-3B-0Y-0SD	Egypt
8	Bani-Suef 7	CBC509CHILE//SOOTY_9/RASCON_37/9/USDA595/3/D67.3RABI//CRA/4/ALO/5/HUI/ YAV_1/6/ARDENTE/7/HUI/YAV79/8/POD_9. CDSS02-Y01233T-0T0PB-0Y-0M-26Y-0Y-0SD.	Egypt

Table S2. Main soil physico-chemical analysis before wheat cultivation at the experimental site.

Depth (cm)	Sand (%)	Silt (%)	Clay (%)	texture	θ_s (%)	LL (%)	DUL (%)	OC (%)	ϱb (g cm^{-3})	Ks (cm h^{-1})
**0-20**	16.0	30.1	53.9	clay	62.7	22.0	40.6	0.8	1.20	0.40
**20-40**	15.5	32.5	52.0	Clay	65.2	21.5	40.5	0.7	1.22	0.38
**40-60**	14.5	37.0	48.5	clay	65.5	21.0	41.2	0.5	1.23	0.31

	pH	Available N (mg kg^{-1})	Available P (mg kg^{-1})	Available K (mg kg^{-1})	OM (%)
**0-20**	8.0	35.2	10.5	620	1.37
**20-40**	7.8	32.5	9.5	602	1.21
**40-60**	8.2	28.9	9.2	585	0.86

θ_s: saturation percentage (%), LL: wilting point (%), DUL: field capacity (%), OC: organic carbon (%), ϱb: bulk density, K_s: saturated hydraulic conductivity, OM: organic matter

Table S3. Estimates of heterosis relative to the better parent (Heterobeltiosis) for all the studied traits under the two irrigation treatments.

Cross	Days to heading		Plant height (cm)		Spike length (cm)		No. of spikelets/ spike	
	**Normal**	**Stress**	**Normal**	**Stress**	**Normal**	**Stress**	**Normal**	**Stress**
P1×P2	-3.06*	-7.84**	4.90*	11.15**	-0.37	7.77*	3.67	4.00
P1×P3	-3.11*	-5.88**	-0.98	4.35	0.75	-11.48**	-1.39	-2.72
P1×P4	-0.95	-4.90**	1.31	7.43**	-10.18**	-4.83	-3.13	-5.65
P1×P5	-1.90	-4.25**	-6.86**	-0.74	-8.24**	-11.46**	2.01	-2.30
P1×P6	-1.90	-6.54**	-3.59	1.82	-13.11**	-5.73	-3.44	-6.10
P1×P7	-1.27	-5.88**	-6.03*	-5.78*	-7.67**	-11.84**	-3.14	-11.64**
P1×P8	-2.64	-2.61	-6.69*	0.74	-4.49	-2.34	-8.10**	-13.64**
P2×P3	3.06*	0.00	2.73	1.09	0.75	7.66*	2.75	11.05**
P2×P4	-3.36*	0.00	2.41	3.36	-4.21	9.66**	6.10	1.59
P2×P5	-4.89**	-0.33	2.44	0.75	5.62*	4.66	4.74	4.36
P2×P6	-1.22	-3.62*	1.35	1.09	0.75	-0.52	3.15	-3.39
P2×P7	1.22	-3.62*	-5.40*	-6.80**	0.33	-8.77**	-2.14	-4.92
P2×P8	-3.36*	-6.91**	-0.70	-2.61	3.37	-9.81**	1.68	-3.25
P3×P4	-2.80*	-1.99	-1.71	-3.62	5.96**	-7.18*	3.42	-2.83
P3×P5	-4.66**	-4.30**	1.37	-1.09	3.57	-7.66*	0.47	-4.71
P3×P6	-2.17	-6.62**	0.67	-0.36	13.10**	-9.57**	2.29	-6.10
P3×P7	-4.35**	-3.97**	-5.08*	-3.74	0.67	-5.26	-2.71	-5.08
P3×P8	-3.42*	-2.32	2.73	0.00	6.37**	-1.40	-5.31	-9.58**
P4×P5	-1.95	-0.35	-0.69	-0.38	-7.37**	-10.14**	-2.83	-8.13*
P4×P6	-0.65	-1.72	1.35	0.73	-3.86	-5.31	-2.01	-3.56
P4×P7	-3.50*	-2.05	-0.63	-1.70	-2.33	-8.77**	-0.57	-9.18**
P4×P8	-4.56**	-2.38	1.72	3.02	-2.11	-11.68**	-12.01**	-9.74**
P5×P6	0.33	-1.72	-4.04	-4.38	0.84	-12.50**	-6.02	-10.85**
P5×P7	-3.82**	-0.34	-6.35**	-6.46**	-4.00	-2.63	-6.42*	-12.46**
P5×P8	3.30*	5.10**	2.46	-2.66	5.62*	4.21	-6.28*	-16.72**
P6×P7	-0.64	-2.74	-5.40*	-7.48**	-8.00**	-8.33**	-0.43	-6.07
P6×P8	-0.98	-5.44**	-2.36	-2.55	0.37	-6.07*	-4.75	-12.01**
P7×P8	-4.55**	-3.06*	-7.30**	-7.82**	-9.67**	-6.14*	-5.59	-9.74**

Table S3. Cont.

Cross	No. of spikes /plant		No. of kernels / spike		1000-kernel weight (g)		Grain yield/plant (g)	
	Normal	Stress	Normal	Stress	Normal	Stress	Normal	Stress
P1×P2	-3.57	-2.08	9.72*	5.70	13.79*	14.39*	34.17**	24.76**
P1×P3	7.02	6.25	-6.88	-7.89	10.34	11.36	15.52**	-0.98
P1×P4	0.00	0.00	-11.16*	-10.73*	6.90	3.79	-0.92	-13.68**
P1×P5	15.91*	18.92*	-20.24**	-17.54**	4.14	4.55	32.99**	39.74**
P1×P6	-1.79	-4.08	-3.80	-9.50*	5.00	-1.33	22.70**	7.83*
P1×P7	-1.52	-20.00*	0.71	-1.54	2.35	-4.40	13.73**	12.50**
P1×P8	11.76	-1.59	7.84	1.61	0.00	-1.82	9.27**	7.38*
P2×P3	-5.26	-6.25	2.46	4.46	12.59*	17.05**	22.50**	7.62*
P2×P4	10.71	10.42	1.20	3.86	3.52	3.91	11.67**	0.00
P2×P5	0.00	-4.17	-3.28	-1.34	8.45	6.25	10.83**	-1.90
P2×P6	12.50	16.33	-1.14	-1.24	6.25	4.67	8.51**	13.91**
P2×P7	1.52	-6.67	3.53	2.31	7.65	6.29	11.11**	22.50**
P2×P8	4.41	-1.59	9.02*	4.02	-6.99	-3.64	9.27**	18.85**
P3×P4	1.75	6.25	-4.38	-4.72	11.19	12.40*	19.83**	8.82*
P3×P5	18.77*	16.67*	9.33*	15.00**	10.49	9.30	24.14**	17.65**
P3×P6	8.77	12.24	6.08	7.85	5.00	0.67	19.15**	12.17**
P3×P7	7.58	0.00	1.77	2.69	4.71	3.77	10.46**	13.33**
P3×P8	2.94	1.59	10.59*	5.22	-5.91	-2.42	7.28*	8.20**
P4×P5	45.65**	43.24**	3.98	5.58	15.71*	15.75*	27.52**	15.79**
P4×P6	7.14	8.16	2.28	7.02	6.25	5.33	22.70**	33.04**
P4×P7	1.52	-3.33	11.31**	6.54	6.47	7.55	13.07**	15.83**
P4×P8	4.41	0.00	14.90**	7.23	-4.84	-2.42	11.26**	14.75**
P5×P6	1.79	4.08	-4.18	-7.85	-5.00	-10.67*	-17.02**	-18.26**
P5×P7	-3.03	-5.00	-0.35	2.31	7.06	6.29	10.46**	9.17**
P5×P8	13.24	4.76	16.86**	4.02	-6.45	-1.82	8.61**	13.11**
P6×P7	0.00	6.67	2.83	6.92	-2.94	-5.66	18.30**	19.17**
P6×P8	11.76	6.35	10.27*	12.05**	-5.91	0.61	15.23**	31.97**
P7×P8	19.12*	15.87*	0.35	6.15	2.69	7.27	18.30**	38.52**

* and ** significant at 0.05 and 0.01 levels of probability, respectively.

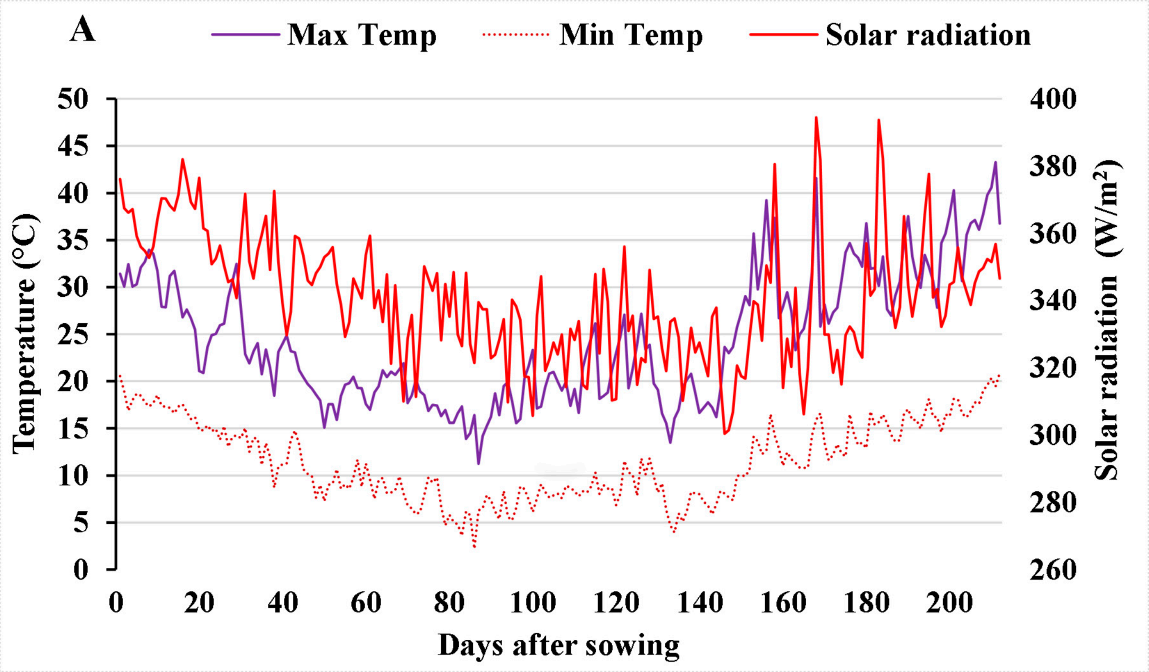

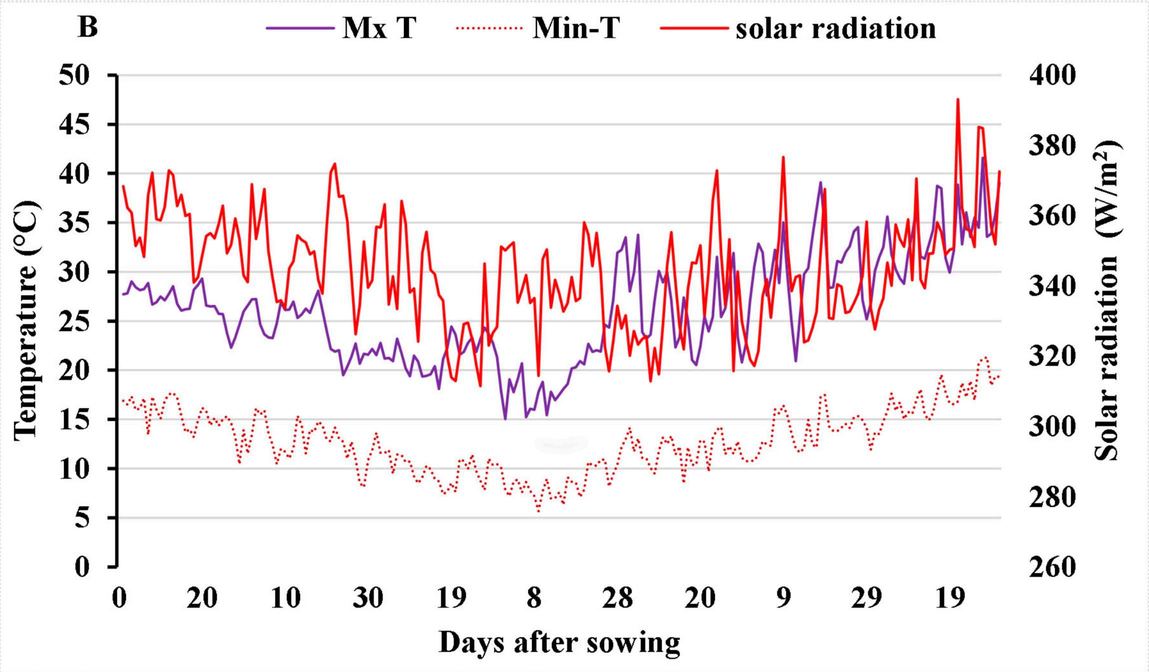

Figure S1. Minimum temperature, maximum temperature and Solar radiation at experimental site during first (A) and second (B) seasons.

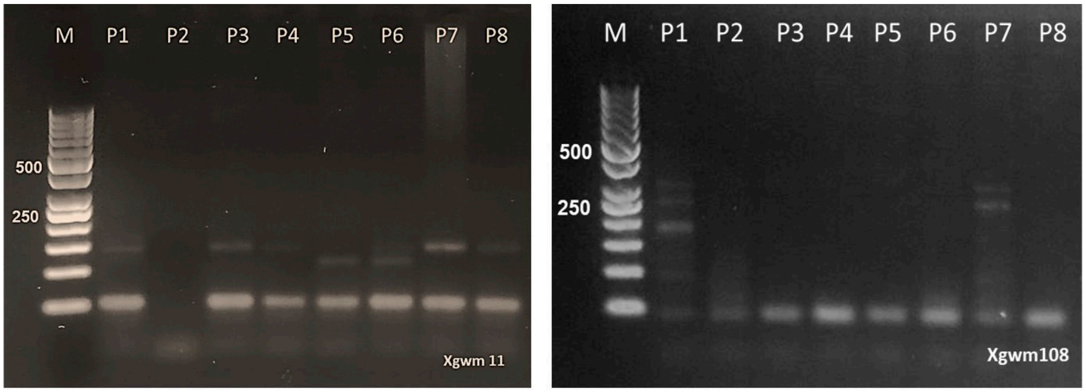

Figure S2. Electrophoresed gel image of the simple sequence repeat (SSR) markers: Xgwm 11 and Xgwm 108.
